# Take 2: The maintenance of a colorectal cancer screening program in intervention clinics and roll out in usual care clinics during the second year of a pragmatic trial

**DOI:** 10.21203/rs.3.rs-9163243/v1

**Published:** 2026-04-19

**Authors:** Amanda F. Petrik, Gloria D. Coronado, Robert Durr, Jennifer Coury, Brittany Badicke, Erin S. Kenzie, Anders Herreid-O’Neill, Anna Edelmann, Emily Myers, Maryan Carbuccia, Melinda M. Davis

**Affiliations:** Kaiser Permanente Center for Health Research; University of Arizona Cancer Center; Oregon Rural Practice-based Research Network; Oregon Rural Practice-based Research Network; Oregon Rural Practice-based Research Network; Oregon Rural Practice-based Research Network; Oregon Rural Practice-based Research Network; Kaiser Permanente Center for Health Research; Oregon Rural Practice-based Research Network; Oregon Rural Practice-based Research Network; Oregon Rural Practice-based Research Network

## Abstract

**Background:**

In the United States, patients in rural settings screen for colorectal cancer (CRC) at a lower rate than urban patients. SMARTER CRC, a pragmatic trial to increase CRC screening in rural clinics in Oregon, implemented a screening program that included 1) mailing fecal immunochemical tests (FIT tests) in partnership with Medicaid health plans and 2) training clinics in navigating patients to complete colonoscopy follow-up to abnormal FIT testing. We describe the second year of the SMARTER CRC project, which includes the second year of implementation for the intervention clinics, and the first year of program rollout for the usual care clinics.

**Methods:**

SMARTER CRC partnered with three Medicaid health plans and 26 affiliated rural clinics. Our implementation outcomes in this mixed methods analysis were the proportion of eligible enrollees who were mailed a FIT and completed any CRC screening or FIT screening in 6 months or 12 months. We also measured if Year 1 clinics were more likely to continue CRC screening (overall and via FIT) at the same level or to increase screening in Year 2. Our qualitative evaluation contextualizes quantitative findings.

**Results:**

In Year 1, Medicaid health plans identified 5,614 enrollees due for screening. After clinic staff review, FIT mailings were sent to 1,489 enrollees in intervention clinics. In Year 2, health plans identified 9,805 enrollees due for CRC screening and mailings were sent to 2,764 in the intervention clinics and 3,505 in the usual care clinics. Overall CRC screening rates and FIT completion rates among eligible patients in the intervention clinics dropped from Year 1 to Year 2 (CRC 19.9% to 12.0%, p value = < 0.00; and FIT 15.7% to 6.6%, p value = < 0.00). In usual care clinics, overall screening increased (9.3% in Year 1 to 15% in Year 2, p-value = < 0.00, and FIT completion increased from 4.9% to 9.6%, p-value = <0.00).

**Conclusions:**

CRC screening rates dropped in the second-year implementation in intervention clinics yet increased in intervention-naïve usual care clinics. Sustaining impacts of effective implementation of FIT outreach interventions over time may require ongoing effort and support.

## INTRODUCTION

Colorectal cancer (CRC) continues to be an unnecessary leading cause of cancer deaths in the U.S. This is largely because CRC screening continues to be underutilized, regardless of multiple effective methods for screening. Individuals in rural settings have higher overall colorectal cancer incidence and screen at lower rates than individuals in urban settings. [1–4]

Mailed FIT programs are successful, based on data from 6-month or 12-month follow-up, but little is known about the sustainment of such programs beyond 12 months.[5] Few studies complete ongoing assessment of screening.[6] Studies have shown that adherence is low because of reduced ordering in the second year, and low adherence among patients who did not screen in the first year.[7] Annual testing even in heavily managed health systems like Kaiser Permanente is low, with a 24% adherence rate over 4 years.[8]

Implementation and dissemination science can help identify effective ways to implement evidence-based interventions to increase screening. These studies reveal how implementation strategies perform across diverse practice settings, such as rural clinics or health systems.[6, 9] In particular, examining the rollout of complex system-level interventions beyond a randomized clinical trial can further uncover adoption barriers, adaptations, and sustainability insights, and is therefore essential to judge real-world effectiveness and scalability.[9, 10]

SMARTER CRC was a large-scale, parallel, two-arm cluster-randomized trial to increase CRC screening in rural clinics in Oregon and is described in detail elsewhere.[5, 11] Briefly, SMARTER CRC implemented a screening program including mailing fecal immunochemical tests (FIT) in partnership with Medicaid health plans, and training clinics in patient navigation to complete colonoscopy follow-up to abnormal FIT testing. In Year 1, Medicaid enrollees in intervention clinics had an adjusted 7.3% higher rate of CRC screening completion than usual care clinics.[5] Only eight of the fourteen Year 1 intervention clinics (57%) had any patients eligible for patient navigation; we found barriers, facilitators, and adaptations to the patient navigation protocol, which are described elsewhere.[12]

Here, we describe the second year of the SMARTER CRC project, which includes the second year of intervention for the intervention clinics, and the first year of intervention rollout for the usual care clinics. We hypothesized that Year 2 implementation in the intervention clinics would be more successful than the first year; and that the usual care clinics rolling out the screening program would be more successful than the intervention clinics were in Year 1 due to study team and health plan experience supporting program implementation.

## METHODS

SMARTER CRC was conducted as part of the National Cancer Institute (NCI)-funded consortium Accelerating Colorectal Cancer Screening and Follow-up through Implementation Science (ACCSIS) Program (NCI Clinical Trials Reporting Program: NCT04890054).[13] SMARTER CRC was a partnership between the Oregon Health & Science University (OHSU)’s Oregon Rural Practice-based Research Network and the Kaiser Permanente Center for Health Research. The OHSU Institutional Review Board approved the trial (protocol number: 20681) and granted a waiver of informed consent as the study involved minimal risks.

SMARTER CRC involved three Medicaid health plans and 26 clinics (i.e., individual clinics or groups of clinics), followed by a scale-up study involving 130 clinics that serve rural counties in Oregon.[14] [11, 15] The research team and participating health plans recruited health plan-affiliated clinics that had at least 30 patients who were age-eligible and had Medicaid insurance or were dually enrolled in Medicaid and Medicare; had CRC screening rates of 60% or lower; and operated in a geographic region designated as rural or frontier.[16, 17],[18] The intervention was tailored for rural Medicaid populations, with components delivered by health plans, clinics, and direct-mail vendors.[19]

In the SMARTER CRC pragmatic trial, clinics were randomized to implement the intervention or continue providing usual care from May 11, 2021, through June 4, 2022. Here, we analyze data from “Year 2” – the year following the randomized trial, when all enrolled clinics were offered the intervention and were eligible to conduct the study activities. The Year 2 intervention was delivered May 18, 2022, through June 4, 2023. We use the term “Year 1 intervention clinic” to designate the clinics assigned to receive the intervention as part of the randomized trial in Year 1, and “Year 1 usual care clinic” to designate a clinic that began implementing the SMARTER CRC intervention in Year 2.

Clinics were randomized by clinic unit affiliation (hospital affiliated, health care network affiliated, multiple locations, or single location) to either usual care (n = 14) or pragmatic intervention (n = 15). Shortly after randomization, one intervention clinic closed (patients were absorbed in other study clinics), leaving 14 intervention clinic units. In Year 2 of the project, following the study design, randomization was rescinded, and all clinics enrolled received the facilitated intervention components.

Practice facilitators from the research team were assigned to each participating clinic to provide tailored implementation support.[11] This included a review of their baseline intake survey to address any notable changes, a workflow assessment for newly implementing clinics (i.e., usual care in Year 2) for program implementation, a workflow reassessment for continuing clinics (e.g., intervention) to address any new adaptations identified, monthly meetings with clinics and health plan staff, supplemental training for clinics experiencing staff turnover, and ad hoc meetings to address clinic needs. Practice facilitators captured detailed field notes about each encounter with their assigned clinics, including mailed FIT and patient navigation progress and challenges, disruptions, adaptations, resources provided, action items, and an assessment of the clinic’s ability to continue to make progress.

Health plan staff used claims data to identify enrollees who were ages 50–75 in Year 1 and 45–75 in Year 2, enrolled in Medicaid or dually enrolled in Medicaid and Medicare, and had no disqualifying health condition based on codes used in prior studies and described in the protocol.[20, 21] Identified enrollees were entered into a REDCap database (Fort Lauderdale, FL) developed for the project.[22] From May to June 2023, clinic staff delivered combinations of introductory letters, kits and instruction sheets, and a preaddressed and postage-paid envelope (all health plans) to all intervention-eligible enrollees.

### Analysis

The primary outcome was receipt of any CRC screening for eligible participants within 12 months of the claims list pull date; we examined whether participants in Year 1 intervention clinics were more likely to continue screening at the same level or increase screening in Year 2. We also measured FIT completion at 12 months and reach by calculating the proportion of eligible enrollees who were mailed and completed a FIT at 12 months.

We report frequency of enrollees’ demographic characteristics in intervention and usual care clinics. Among intervention clinics, we report first- and second-year numbers of eligible enrollees, numbers scrubbed by clinic staff, numbers mailed a FIT test, and proportions that obtained CRC screening, and FIT testing, within 12 months. We report differences in first and second year proportions of enrollees who were mailed a FIT, screened for CRC, or completed a FIT test at 12 months. Similarly, for usual care clinics, we report differences in first and second year outcomes. We also report the results of bivariate analysis using χ2 test of difference for categorical variables including CRC screening at 12 months and FIT screening at 12 months to assess the change in screening from Year 1 to Year 2 overall, by clinic, and by health plan.

### Qualitative Data Collection and Analysis

Qualitative data was collected at several points during the program. These included interviews with staff at participating clinics and health plans at four time points: baseline, following completion of the first year, following completion of the second year, and oneyear post-intervention. Supplemental interviews were completed with patient navigators at clinics following first-year implementation. Interviews were carried out by trained qualitative analysts using semi-structured interview guides [Additional file 1].

Practice facilitators also logged field notes about clinic interactions into the REDCap database; these included summaries of ad hoc communications and planned program meetings occurring across both implementation years. More detailed descriptions of data collection and analytic approach have been described previously.[11] Practice facilitators and other program staff participated in regular periodic reflections led by a qualitative analyst, who collected data on team perceptions of facilitators and barriers, differences between clinic implementations, and relevant program milestones.[23]

Qualitative data was transcribed, cleaned, and uploaded to ATLAS.ti 23.4, a qualitative data analysis program.[24] Codebooks were developed by the team based on research questions, topic-area expert input, and additional open coding. Transcripts were co-coded by qualitative team members and compared to establish coder agreement; all data was then split between coders and coded. Code query reports were pulled, and an immersion crystallization analysis approach[25] was used to identify themes (barriers, facilitators, year comparison). A subsection of each code report was analyzed by multiple analysts to ensure agreement.

## RESULTS

### Quantitative Results

**Figure 1:**
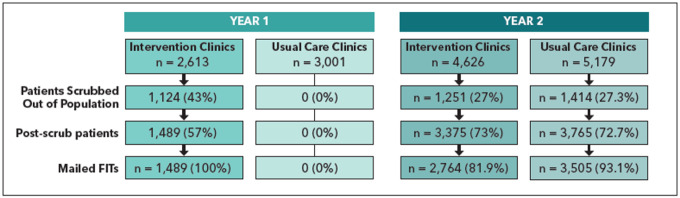
Consort Diagram

In Year 1, three Medicaid health plans identified 5,614 patients due for CRC screening (Fig. 1, n = 2,613 in intervention Clinics, n = 3,001 in usual care clinics). The health plans sent the list to the intervention Clinics for review and 43% (n = 1,124) were scrubbed out due to no longer being a clinic patient, not having established care with the clinic, or having already completed screening. Mailings were sent to 100% of the remaining 1,489 patients. In Year 2, health plans identified 9,805 patients due for CRC screening. Two intervention Clinics did not participate in Year 2. Health plans sent the list to both intervention and usual care clinics for review and 27% (n = 1,251) and 27.3% (n = 1,414) were scrubbed out, respectively. Of the remaining patients, mailings were sent to 81.9% (n = 2,764) in the intervention clinics and 93.1% (n = 3,505) in the usual care clinics.

To compare the first year of implementation in each arm, we report on the demographic characteristics of 2,613 eligible enrollees in 14 intervention Clinics in Year 1 and 5,179 eligible enrollees in 14 usual care clinics in Year 2 (Table 1.). Overall, clinic and enrollee characteristics were similar across intervention and usual care groups. Intervention patients in general were younger, were more likely to be White (77% vs. 58.4%), their clinics were more rural, and more intervention patients had prior CRC screening. Roughly 54% in both groups were female, less than 4% were Hispanic ethnicity, and at least 97% had English listed as their preferred language. Usual care and intervention clinics had few isolated rural patients (6.3% and 4.9% respectively). According to the Office of Rural Health classification, intervention clinics had a median of 97.1% of patients lived in rural areas, and usual care clinics had median of 86.5% of patients who lived in rural areas, however the percentage of rural patients in each clinic ranged from 0–100%.[16]

Overall CRC screening rates and FIT completion rates among eligible enrollees in the intervention clinics dropped from Year 1 to Year 2 (CRC 19.9% to 12.0%, p value = < 0.00; and FIT 15.7% to 6.6%, p value = < 0.00; respectively; Table 2.). Results varied by health plan with CRC screening rates ranging from a 4.7% drop in Health Plan 1 to a 13.6% drop in Health Plan 2. FIT completion rates dropped also by health plan, ranging from a 7.0% overall drop in Health Plan 3 to a 14.7% drop in Health Plan 2. In usual care clinics, overall screening increased after implementation from 9.3% in Year 1 to 15.0% in Year 2 (p value = < 0.00), and FIT completion increased from 4.9% to 9.6% (p value = < 0.00). Results also varied by health plan, ranging from a 3.7%−6.6% increase in CRC screening, and a 3.7%−6.9% increase in FIT completion.

Fewer enrollees were scrubbed out across all health plans in Year 2 than in Year 1 (27% in Year 2 vs. 43% in Year 1). Fewer usual care patients in Year 2 were also scrubbed (27.3%). The proportion of eligible enrollees who were mailed a FIT decreased compared to the intervention clinics 100% mailing in Year 1; Year 2 intervention clinics mailed to 81.9%, and Year 2 usual care clinics mailed to 93.1% of eligible enrollees.

**Figure 2:**
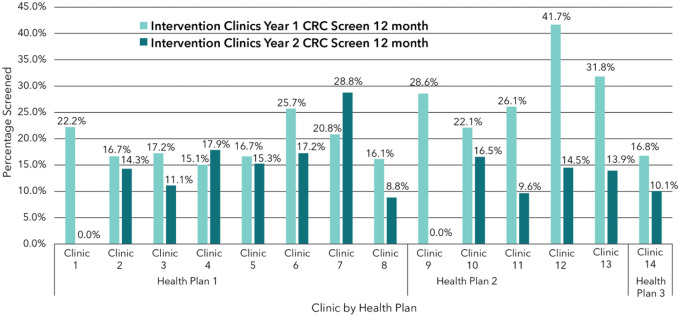
Intervention Clinics Year 1 vs. Year 2 CRC Screening at 12 Months

**Figure 3:**
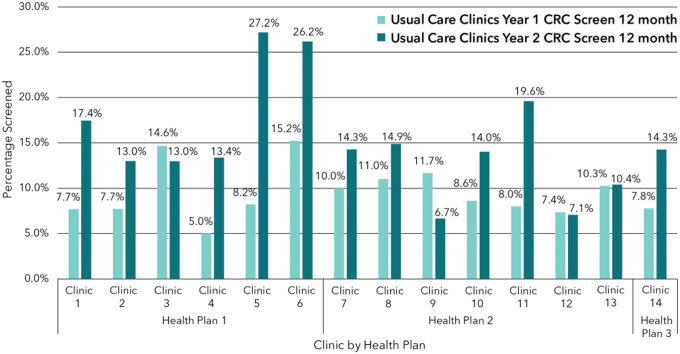
Usual Care Clinics Year 1 vs. Year 2 CRC Screening at 12 Months

Individual intervention Clinic rates of overall CRC screening varied. While two intervention clinics saw an increase from Year 1 to Year 2 (clinics 4 and 7), the remaining clinics saw decreased screening completion among eligible patients from Year 1 to Year 2 (Fig. 2). Usual care success rates also varied, with 3 clinics (3, 9, and 12) decreasing overall screening after implementation, and the remaining 11 clinics increasing screening (Fig. 3).

### Qualitative Results

Throughout the study, clinics and health plans experienced a variety of challenges to implementation of the SMARTER CRC intervention, with staffing shortages and turnover primary among them. Study recruitment and implementation took place during the COVID-19 pandemic, which compounded workforce challenges and presented urgent pressures on primary care staff to care for ill patients and participate in vaccination efforts. Clinics also reported internal organizational challenges, such as miscommunication, shifting of roles among staff, and differing expectations about study participation. Practice facilitators described offering additional support to clinics when they were aware of clinic staff turnover or reassignment, although the uptake of that support varied and facilitators were often not informed of turnover. Year 1 Intervention clinics were excluded from some of the early Year 2 health plan-clinic meetings about program basics; facilitators later expressed that the clinics might have benefited from the review. Facilitators did report offering more individualized support to clinics in Year 2.

Prior to the program’s second year, two intervention clinics dropped out of the SMARTER CRC program. In baseline interviews, staff at both clinics described having CRC champions and that they were able to complete the Year 1 scrubbing and mailing. However, both clinics experienced team lead turnover during the first year of implementation. Reasons for dropping out included that the SMARTER CRC project was a low priority, and COVID-related staffing issues. Low FIT return rates and patient resistance may have further lowered the program as a priority during a challenging time for rural clinics overall.

Challenges were also faced by health plan partners, particularly in the second year of implementation. In Year 2, health plans experienced delays in FIT mailing due to one health plan’s FIT mailing vendor and another health plan’s new process for ordering FITs from the lab. One clinic cited this delay as a contributing factor to their lower overall screening rates in Year 2. They felt their clinic delayed typical CRC outreach while waiting for the FIT mailing. Health plans also had difficulty generating accurate lists of eligible patients, requiring additional support from the study team. One health plan experienced a change in the study’s primary point of contact midway through the study.

## DISCUSSION

In the second year of the SMARTER CRC project, CRC screening rates decreased from Year 1 to Year 2 in intervention clinics, and implementation was less successful among the usual care clinics compared to Year 1 rollout in the intervention clinics. Several factors could explain this finding, including a large increase in eligible enrollees in Year 2 because of the lowering of the screening age to 45, and changes in the Medicaid redetermination rules during the pandemic[26], differences in clinic staff review of enrollee lists, mailing delays and changes in vendors that mailed FIT components. Sustaining impacts of effective implementation programs to increase CRC screening by FIT outreach over time will require ongoing effort and support.

It may be informative to consider the differences in program effectiveness across health plans. The health plans chose implementation strategies (i.e., reminders, vendor) with the support of the study team, which may explain some variation in program effectiveness. However, in general, rural practice settings experience low FIT return rates, and the return rates we saw in our study fit with that pattern. The highest return rate among the health plans was 21.9% in Year 1 intervention clinics, but across all Year 1 intervention clinics the FIT return rate was 15.7%, and the FIT return rate was 9.6% in Year 2 usual care clinics. Future studies should seek to understand why rural patients are consistently less likely to screen and to return FITs.[27]

There was also limited scrubbing and a delay in mailings in Year 2. One intervention clinic and 1 usual care clinic did not scrub at all in Year 2. Higher clinic scrub rates in Year 1 intervention clinics could explain increased screening rates in clinics that effectively removed patients from the mailing list. Additionally, there were delays in mailings across all health plans in Year 2, especially in Health Plan 2 because of a change in mailing vendor. The health plan delays and communication issues between clinics and health plans may have been caused by the scale of this large pragmatic study; delays may not have been experienced if the clinic was working independently with the health plan. Moreover, although clinics were not instructed to pause usual CRC outreach, the FIT mailing delay in Year 2 may have had this unintended consequence. A more pragmatic approach to the mailing in general could help with some of the mailing delays and challenges as well as a clear message that mailed programs are intended to complement in-clinic activities rather than replace them.

Reduced screening in the intervention clinics could also be due to having more screeningresistant patients eligible in Year 2 compared to Year 1 because “screening likely” patients were screened in Year 1. The Year 1 intervention clinics could have “reached” all the patients that would have returned a FIT in Year 1. The design of the intervention (each year) may have unintentionally excluded the most likely re-screeners, as they would not have yet been due for screening by the second mailing. Future mail-out programs should include both previous completers (“early screeners”) and new participants. The design of implementation programs in subsequent years should include planned follow-up for Year 1 screeners, as well as the ongoing unscreened population.

Alternatively, Year 1 may have been more successful due to program implementation expertise. The project team assumed that the Year 1 intervention clinics would require less guidance and offered reduced facilitation support as the usual care clinics needed more support. However, ongoing support for clinics who implemented the previous year appears critical. In addition, sustainability will require institutionalization of the program within health plan and clinic workflows, not reliance on short-term research facilitation.

We learned from this project that rural clinics and health plans did not develop adequate capacity for a successful program in one year. It is possible that small rural clinics need assistance with growing their workforce and staffing for programmatic implementation projects. Clinics may need further assistance and reimbursement to build sustainable programs.

This study has limitations, including that this study was conducted in one state (Oregon) and only with three health plans. Further, we do not report on follow-up to abnormal testing due to the inability to conduct chart reviews in Year 2 of the study.

### Conclusion

CRC screening rates overall and FIT screening dropped in the second year of the SMARTER CRC study, yet CRC screening rates overall and by FIT increased in intervention-naïve usual care clinics. Sustaining impacts of effective implementation of FIT outreach interventions over time may require ongoing effort and support.

## Supplementary Files

This is a list of supplementary files associated with this preprint. Click to download.

- Additionalfile1ClinicExitInterviewFinal.docx
- Tables.docx

Tables 1 and 2 are available in the Supplementary Files section.

## Data Availability

Data from this manuscript is available from the corresponding author upon reasonable request.

## References

[1] Sabatino SA, White MC, Thompson TD, Klabunde CN. Cancer screening test use - United States, 2013. MMWR Morb Mortal Wkly Rep. 2015;64(17):464–8. 25950253 PMC4584551

[2] Cole AM, Jackson JE, Doescher M. Urban-rural disparities in colorectal cancer screening: cross-sectional analysis of 1998–2005 data from the Centers for Disease Control’s Behavioral Risk Factor Surveillance Study. Cancer Med. 2012;1(3):350–6. 23342284 10.1002/cam4.40 PMC3544460

[3] Henley SJ, Anderson RN, Thomas CC, Massetti GM, Peaker B, Richardson LC, Invasive Cancer Incidence. 2004–2013, and Deaths, 2006–2015, in Nonmetropolitan and Metropolitan Counties - United States. Morbidity and mortality weekly report Surveillance summaries (Washington, DC: 2002). 2017;66(14):1–13. 10.15585/mmwr.ss6614a1 PMC5879727 28683054

[4] Semprini J, Gadag K, Williams G, Muldrow A, Zahnd WE. Rural-Urban Cancer Incidence and Trends in the United States, 2000 to 2019. Cancer Epidemiol Biomarkers Prev. 2024;33(8):1012–22. 38801414 10.1158/1055-9965.EPI-24-0072

[5] Coronado GD, Petrik AF, Leo MC, Coury J, Durr R, Badicke B, Mailed Outreach and Patient Navigation for Colorectal Cancer Screening Among Rural Medicaid Enrollees: A Cluster Randomized Clinical Trial. JAMA Netw Open. 2025;8(3):e250928–e. 40094661 10.1001/jamanetworkopen.2025.0928 PMC11915063

[6] Dougherty MK, Brenner AT, Crockett SD, Gupta S, Wheeler SB, Coker-Schwimmer M, Evaluation of Interventions Intended to Increase Colorectal Cancer Screening Rates in the United States: A Systematic Review and Meta-analysis. JAMA Intern Med. 2018;178(12):1645–58. 30326005 10.1001/jamainternmed.2018.4637 PMC6583619

[7] Nielson CM, Vollmer WM, Petrik AF, Keast EM, Green BB, Coronado GD. Factors Affecting Adherence in a Pragmatic Trial of Annual Fecal Immunochemical Testing for Colorectal Cancer. J Gen Intern Med. 2019;34(6):978–85. 30684199 10.1007/s11606-018-4820-0 PMC6544723

[8] Lin JS, Perdue LA, Henrikson NB, Bean SI, Blasi PR. Screening for Colorectal Cancer: Updated Evidence Report and Systematic Review for the US Preventive Services Task Force. JAMA. 2021;325(19):1978–98. 34003220 10.1001/jama.2021.4417 PMC13509038

[9] Adsul P, Kanabar N, Kruse-Diehr A, Dignan M, Oliveri JM, Paskett ED, Generating the evidence base for implementation strategies targeting colorectal cancer screening in the accelerating colorectal cancer screening through implementation science (ACCSIS) research projects. BMC Public Health. 2026;26(1):576. 41580763 10.1186/s12889-025-26179-2 PMC12896049

[10] Shelton RC, Hailemariam M, Iwelunmor J. Making the connection between health equity and sustainability. Front Public Health. 2023;11:1226175. 37822544 10.3389/fpubh.2023.1226175 PMC10562623

[11] Coronado GDLM, Ramsey K, Coury J, Petrik AF, Patzel M, Kenzie E, Thompson JH, Brodt E, Mummadi R, Elder N, Davis MM. Mailed fecal testing and patient navigation versus usual care to improve rates of colorectal cancer screening and follow-up colonoscopy in rural Medicaid enrollees: A cluster-randomized controlled trial. 2022. 10.1186/s43058-022-00285-3 PMC9006522 35418107

[12] Myers E, Coury J, Carbuccia-Abbott M, Petrik AF, Durr R, Thompson JH, Qualitative Outcomes of Colorectal Cancer Screening Outreach Using Patient Navigation to Follow-Up Colonoscopy in Rural Primary Care Practices. Cancers (Basel). 2025;17:21. 10.3390/cancers17213590 PMC12609902 41228381

[13] RTI International. Accelerating Colorectal Cancer Screening and Follow-Up Through Implementation Science (ACCSIS) [December 16, 2024]. Available from: https://accsis.rti.org/

[14] Gupta S, Coronado GD, Argenbright K, Brenner AT, Castañeda SF, Dominitz JA, Mailed fecal immunochemical test outreach for colorectal cancer screening: Summary of a Centers for Disease Control and Prevention-sponsored Summit. CA Cancer J Clin. 2020;70(4):283–98. 32583884 10.3322/caac.21615 PMC7523556

[15] Davis MM, Coury J, Larson JH, Gunn R, Towey EG, Ketelhut A, Improving colorectal cancer screening in rural primary care: Preliminary effectiveness and implementation of a collaborative mailed fecal immunochemical test pilot. J Rural Health. 2023;39(1):279–90. 35703582 10.1111/jrh.12685 PMC9969840

[16] Oregon Office of Rural Health. Oregon Office of Rural Health Geographic Definitions 2021 [2021]. Available from: https://www.ohsu.edu/oregon-office-of-ruralhealth/about-rural-and-frontier-data

[17] USDA Economic Research Service. Rural-Urban Commuting Area Codes 2020 [2022]. Available from: https://www.ers.usda.gov/data-products/rural-urban-commuting-area-codes.aspx

[18] ClinicalTrials.gov. Screening More Patients for Colorectal Cancer Through Adapting and Refining Targeted Evidence-Based Interventions in Rural Settings, SMARTER CRC (SMARTER CRC) https://clinicaltrials.gov/ct2/show/NCT048900542022 [02/22/2022]

[19] Coronado GD, Johnson ES, Leo MC, Schneider JL, Smith D, Mummadi R, Patient randomized trial of a targeted navigation program to improve rates of follow-up colonoscopy in community health centers. Contemp Clin Trials. 2020;89:105920. 31881390 10.1016/j.cct.2019.105920 PMC7254876

[20] Coronado GD, Vollmer WM, Petrik A, Taplin SH, Burdick TE, Meenan RT, Strategies and Opportunities to STOP Colon Cancer in Priority Populations: design of a cluster-randomized pragmatic trial. Contemp Clin Trials. 2014;38(2):344–9. 24937017 10.1016/j.cct.2014.06.006 PMC4226652

[21] GD C. Mailed FIT outreach to improve CRC screening 2022 [cited 2022 March 26]. Available from: www.mailedFIT.org.

[22] Harris PA, Taylor R, Minor BL, Elliott V, Fernandez M, O’Neal L, The REDCap consortium: Building an international community of software platform partners. J Biomed Inform. 2019;95:103208. 31078660 10.1016/j.jbi.2019.103208 PMC7254481

[23] Finley EP, Huynh AK, Farmer MM, Bean-Mayberry B, Moin T, Oishi SM, Periodic reflections: a method of guided discussions for documenting implementation phenomena. BMC Med Res Methodol. 2018;18(1):153. 30482159 10.1186/s12874-018-0610-y PMC6258449

[24] ATLAS.ti. Scientific Software Development GmbH 2023. Available from: https://atlasti.com.

[25] Borkan JM. Immersion-Crystallization: a valuable analytic tool for healthcare research. Fam Pract. 2022;39(4):785–9. 34849754 10.1093/fampra/cmab158

[26] Khorrami P, Sommers BD. Changes in US Medicaid Enrollment During the COVID-19 Pandemic. JAMA Netw Open. 2021;4(5):e219463. 33950210 10.1001/jamanetworkopen.2021.9463 PMC8100862

[27] Sepassi A, Li M, Zell JA, Chan A, Saunders IM, Mukamel DB. Rural-Urban Disparities in Colorectal Cancer Screening, Diagnosis, Treatment, and Survivorship Care: A Systematic Review and Meta-Analysis. Oncologist. 2024;29(4):e431–46. 38243853 10.1093/oncolo/oyad347 PMC10994268

